# Recollections of Hospital Life

**Published:** 1887-12-31

**Authors:** William J. L. Hooper

**Affiliations:** Croydon


### THE EDITOR'S LETTER-BOX.

[Our Correspondents are reminded that prolixity is a great bar to publication, and that brevity of style and conciseness of statement greatly facilitate early publication.]

**Recollections of Hospital Life.**

I can strongly support your correspondent in his truthful account of hospital life. I too owe my life, both to the skill of the surgeons and the kindness of the nurses. It is deplorable and to be deplored that many hospitals are compelled to close their wards for want of funds. I earnestly hope that when the present great depression of trade has passed away, many more friends will come to the front and support by subscriptions some of the noblest institutions of the nineteenth century. It is a grand thing to be able to earn one's own living, but I wonder how many persons at this present moment would have been inmates of workhouses if it had not been for the skill of hospital surgeons. I noticed that your correspondent only appears to speak of one hospital, but I have been an inmate of a very large number of London and country hospitals, and for the life of me I cannot find fault with any. I could tell many pleasant stories about nurses and their kindness to poor patients, but knowing your space is limited I refrain. The life of a hospital nurse is not a light one; their wages, too, are far from being high. All honour to them for their kindness to the suffering poor. I feel sure that the following lines represent the majority of our noble Sisters of Mercy and their assistant nurses:—

"I live for those who love me,
For those who know me true,
For the Heaven that shines above me,
And awaits my spirit too.

"For the wrong that needs resistance,
And the poor who need assistance,
For the future in the distance
And the good that I can do."

Croydon, WILLIAM J. L. HOOPER.

Dec. 13th, 1887.

